# Protective role of *Brucella abortus* specific murine antibodies in inhibiting systemic proliferation of virulent strain 544 in mice and guinea pig 

**DOI:** 10.14202/vetworld.2018.794-799

**Published:** 2018-06-13

**Authors:** Suman Verma, Mayank Rawat, Sanjay Kumawat, Salauddin Qureshi, Gulam Mohd, Ashok Kumar Tiwari

**Affiliations:** 1 Division of Biological Standardization, Indian Veterinary Research Institute, Izatnagar - 243 122, Uttar Pradesh, India; 2 Division of Pharmacology and Toxicology, Indian Veterinary Research Institute, Izatnagar - 243 122, Uttar Pradesh, India

**Keywords:** brucellosis, humoral immunity, mice, passive protection, protective index

## Abstract

**Aim:**

The major objective of the investigation was to evaluate the hitherto uncharacterized potential of *Brucella*-specific antibodies to win the battle against virulent *Brucella* *abortus* infection.

**Materials and Methods:**

*Brucella*-specific immune serum was raised in mice. The antibody titer of serum was determined by standard tube agglutination test and indirect enzyme-linked immunosorbent assays (iELISA). Groups of mice and guinea pigs were passively immunized with serum containing specific agglutinin titers. 24 h after immunization, all animals along with unimmunized controls were challenged with *B. abortus* S544. Total *B. abortus* S544 counts in the spleen of each animal collected on the 7^th^ day of challenge was determined to evaluate the protective index (PI) of anti-*Brucella* serum by statistical analysis.

**Result:**

A dose-dependent protective response to immune mice serum was observed in both experimental models though the values of PI of mice were higher than those obtained for guinea pigs. The PI values in mice passively immunized with 50 IU or 25 IU antibodies were 1.38 and 0.69, respectively. In guinea pigs, however, animals passively immunized with 50 IU or 25 IU antibodies showed PI values equivalent to 0.79 and 0.41, respectively.

**Conclusion:**

The observations support our hypothesis that the presence of antibodies inhibits the initial multiplication and eventual colonization of systemic organs by *B. abortus*. Therefore, a predominant antibody-mediated response induced by a vaccine is expected to protect the animal against the most severe clinical outcome of infection.

## Introduction

*Brucella abortus* is one of the most common zoonotic microbes that affect both bovine and human population. It is Gram-negative, alphaproteobacteria that impart an enormous burden on the Indian economy due to abortion, infertility, reduced calf, and milk production in livestock and also pose a serious impact on public health [1]. Recently, the WHO considered this devastating disease as “forgotten neglected zoonosis,” because of its existence at some national and international level [2]. As per zoonotic importance concern, human brucellosis has high incidence rate as approximately 500,000 new cases reported annually while the prevalence rate exceeds 10/100,000 in some developing countries [3,4]. In human, the disease is characterized by an acute undulating fever with non-specific symptoms such as night sweats, asthenia, insomnia, anorexia, headache, and finally, follow-up a chronic infection potentially displaying endocarditis, meningitis, and osteomyelitis [5,6]. Although domestic animal chiefly harbors the infection, a number of wildlife species also equally contribute the disease by acting as reservoirs [7].

Complete eradication of brucellosis is a challenging task because of unimplementation of eradication policies, especially in developing countries [8]. Vaccination is an only determinant strategy to reduce the disease incidence, especially in developing countries. Different vaccines including live *B. abortus* S19 and RB51 and inactivated 45/20 have been employed for immunization of cattle, but none of these can be considered an ideal preparation because of several limitations [9]. Thus, identification of host-bacterial interaction, host immunology, and pathogen biology could be applicable to serve as a platform to design better biologicals [10,11].

*B. abortus* being considered as an intracellular pathogen, it is overemphasized that the immune protection against clinical *Brucella* infection is mainly orientated toward cell-mediated immunity (CMI) and a vaccine candidate must necessarily induce a predominant CMI response [12]. However, observations on the currently available vaccines against brucellosis as well as other viral vaccines do not confirm the predominant role of CMI in protection [13,14]. In addition, experimental data on the passive transfer of specific CD4+ and CD8+ cells in mice do not conclusively prove the role of CMI [15]. Development of novel vaccines against brucellosis requires a thorough understanding of the specific mechanisms of protection against clinical brucellosis.

On the basis of previous studies, it was hypothesized that the presence of vaccine-induced anti*-Brucella* antibodies not only inhibits post-phagocytic intracellular survival of the organism but also checks their spread to the target organs of the host [16]. Hence, the main objective of this study was to evaluate the uncharacterized potential of immune serum against virulent *B. abortus* infection.

## Materials and Methods

### Ethical approval

All the experimental protocols carried out on laboratory animals were approved (No.F.26-1/2015-16/J.D (R)) by the Animals Ethics Committee (AEC), Joint Director of Research, Indian Veterinary Research Institute (IVRI), Izatnagar - 243 122, India. Forty mice and six guinea pig were procured from the Laboratory Animal Resource Section of IVRI, kept in AEC approved facilities, and received water and food *ad libtum*. For retro-orbital bleeding, mice were anesthetized using chloroform. For euthanization, cervical dislocation method was used for mice while guinea pigs were sacrificed by carbon dioxide asphyxiation [17].

### Bacterial strains

*B. abortus* strain 19 (S19), strain 99 (S99), and strain 544 (S544) were obtained from *Brucella* Referral Laboratory, Division of Veterinary Public Health, IVRI. Both the strains were maintained at 4°C on *Brucella* agar slants after confirmation by morphological, biochemical, and serological examinations. Before experimentation, suspension of each strain having the desired viable count was prepared and stored at 4°C.

### Production of anti-*Brucella* antibodies

To raise anti-*Brucella* antibodies, a group of 20 Swiss albino mice (6-8 weeks’ age, 20±2 g) were immunized with *B. abortus* S19 (10^5^ colony forming units [CFU]/ml), subcutaneously as per the standard protocol [18]. Blood samples were drawn at 2^nd^, 3^rd^, 4^th^, and 5^th^ weeks after to collect immune sera (IS) and stored at −80°C till use. Anti-*Brucella* antibody titer of the pooled antiserum was determined by standard tube agglutination test [19], while the level of immunoglobulin M (IgM) and immunoglobulin G (IgG) was determined by indirect enzyme-linked immunosorbent assays (iELISA) employing isotype-specific conjugates, and result was interpreted in terms of P/N ratio.

### Smooth lipopolysaccharide (SLPS) extraction

The SLPS was extracted from *B. abortus* S99 cells employing an optimized method based on hot phenol-water extraction [20]. Briefly, 5 g of lyophilized cells of *B. abortus* strain 99 was resuspended in 170 ml of distilled water and heated to 66°C. An equal volume of phenol (90% v/v) was added with constant agitation for 20 min. Following cooling, the suspension was centrifuged at 12,000 g for 20 min at 4°C. The phenol layer was filtered to remove cellular debris. Subsequently, three parts of chilled methanol containing 1% (v/v) methanol saturated with sodium acetate were added and left it for 2 h at 4°C. The resulting precipitate was removed by centrifugation at 12,000 g and resuspended in 80 ml distilled water and stored for overnight at 4°C with stirring. The crude SLPS was recovered by centrifugation at 10,000 g for 15 min at 4°C. The resulting pellet was resuspended in 80 ml distilled water and stirred for 1 h. The supernatant was pooled and filtered (0.22 µm), and 50-100 mg each of ribonuclease, deoxyribonuclease, and proteinase K was added. The resulting suspension was incubated at −20°C for 18 h and re-precipitated with methanol as above. Finally, the pellet was resuspended in 2 ml of distilled water and dialyzed against water and freeze-dried and stored at −20°C for future use.

### iELISA

The IgM and IgG levels in 2^nd^, 3^rd^, 4^th^, and 5^th^ weeks’ mice serum were determined by employing the modified Briggs and Skeeles method [21]. All the reagents used for ELISA were optimized by chequerboard method. Pre-immunization mice serum was used as negative control. Briefly, polystyrene 96 well plates (Thermo Fisher Scientific) were passively coated with 100 µl of 1:64 diluted *B. abortus* S99 SLPS and incubated at 4°C overnight. Next day, blocking was done by 5% skim milk prepared in phosphate-buffered saline (PBS) with 0.05% Tween-20 (PBST) for 1 h. Following incubation, the plate was washed thrice with PBST and test antibody (1:64) dilution was added in duplicates in each well of test panel and control panel. Negative serum (1:64) was added in negative control panel. After 1 h incubation, the plate was washed thrice with PBST and goat anti-mouse IgG or IgM HRPO conjugate (1:6000) (Sigma Aldrich, USA) was added and incubated for 1 h. Substrate containing OPD and H_2_O_2_ was added. After 10 min, the reaction was stopped using 1 M H_2_SO_4_, and OD was taken by an ELISA Reader (ASYS Hitech, Austria) at 492 nm. The antibody titer of individual serum sample was expressed as positive/negative (P/N) ratio.

### Homologous passive protection test

To determine the protective efficacy of immune serum in homologous animal model, female Swiss albino mice (n=18) were randomly distributed into three experimental groups consisting of six mice in each. Each mouse in Groups I and II was passively immunized with 25 IU and 50 IU serum (s/c), respectively, while each mouse in Group III as control group was injected with equal volume of PBS. Just after 24 h, all three groups were challenged intraperitoneally (i/p) with 2×10^5^ CFU of *B. abortus* S544 culture. After 7 days, all the mice from each group were sacrificed and spleen was excised aseptically in biosafety cabinet and weighed following recommended procedures [17] and processed for total viable count (TVC) of S544 in each spleen [18]. Briefly, spleen was homogenized individually for 9 times to weight of spleen in buffered saline solution (BSS) (pH 6.8), and three 10-fold dilutions were made (1/10, 1/100, and 1/1000) in the BSS and 100 µl of each dilution was seeded on *Brucella* agar in quadruplicate, incubating 2 plate aerobically, while 2 plates kept in 5% CO_2_ for 4-5 days. Following the enumeration of bacterial colonies, TVC per spleen was determined first as X. In absence of any colony on brucella plates corresponding to the 1/10 dilution, it was considered that spleen was infected with five bacteria. Mean protective response (Y) was calculated after the following transformation: Mean protective response (Y)=log (X/log X), while the protective index (PI) was calculated as mean protective response of control−mean protective response of passively immunized group.

### Heterologous passive protection test

To evaluate the potency of immune serum in guinea pig as heterologous animal model, adult guinea pigs weighing 400-500 g (n=6) were randomly distributed into three experimental groups consisting of two guinea pig in each. Each guinea pig in Groups I and II was passively immunized with 25 IU and 50 IU serum (s/c), respectively, while each guinea pig in Group III as control group was injected with equal volume of PBS. Just after 24 h, all three groups were challenged i/p with 5000 CFU of *B. abortus* S544 culture. After 7 days, all guinea pigs were sacrificed by carbon dioxide asphyxiation and spleens were removed aseptically and processed similarly as in mice. Mean protective response and PI were calculated.

### Spleen weight index (SWI)

It is an indirect way to correlate the protection. SWI of mice and guinea pig was determined by the following formula [18]:

SWI=Spleen weight/Body weight of animal under trial×100.

### Statistical analysis

The data were analyzed using one-way analysis of variance following Tukey test. Significance was defined as *p<0.05, **p<0.01, and ***p<0.001.

## Results

The anti-*Brucella* agglutinin titer of pooled IS was found to be 212 IU/ml. The IgM and IgG titers of IS were also determined by iELISA. The *B. abortus* S99 LPS (0.1 mg/ml) and test antibodies (IS) were used at dilution of 1:64, while conjugates were used at 1:6000 dilutions. Classical IgM immune response was observed as gradual increase in IgM titer from the 2^nd^ week, reached highest peak at the 4^th^ week, and then declined at the 5^th^ week. In case of IgG, gradual increase in titer was observed, but peak response was found at the 5^th^ week with P/N titer 4.61 (Table-1).

**Table-1:** Immune serum IgM and IgG level (titer in P/N ratio of OD at 492) in mice.

Time of bleeding	IgM (mean±SD)	IgG (mean±SD)
2^nd^ week	1.671±0.122^af^	1.4140±0.095^ab^
3^rd^ week	2.0011±0.216^b^	1.8268±0.119^b^
4^th^ week	2.0820±0.039^bc^	3.3947±0.255^c^
5^th^ week	1.5600±0.076^ad^	4.6148±0.399^d^

Mean value bearing different superscripts (a, b, c, and d) in each column differ significantly (p<0.05). IgM=Immunoglobulin M, IgG: Immunoglobulin G, SD=Standard deviation

**Figure-1:**
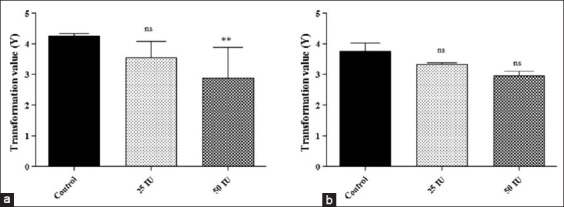
The mean transformation value of total viable spleen count of mice (a) and guinea pig (b) after challenge with *Brucella abortus* S544. Data are presented as mean±standard deviation. NS=Not-significant, (**)=p<0.01.

The protective efficacy was evaluated in terms of mean protective response and PI. There was a significant reduction in total spleen count of passively protected mice or guinea pig as compared to respective control. The mean transformation value of 2.87±1.01, 3.56±0.52, and 4.26±0.07 was found in 50 IU, 25 IU passively protected, and control mice group, respectively (Figure-1). In guinea pig, it was 2.95±0.15, 3.33±0.04, and 3.74±0.29 for 50 IU, 25 IU passively protected, and control group, respectively (Figure-1). By these findings, it can be concluded that serum antibodies could efficiently able to protect against brucellosis. However, comparatively mice were strongly protected than guinea-pig, as 1.38 and 0.69 value of PI was found in mice received 50 IU and 25 IU anti-*Brucella* serum, respectively (Table-2). While in heterologous passive protection test, the PI was 0.79 and 0.41 for guinea pig received 50 IU and 25 IU anti-*Brucella* serum, respectively (Table-3).

**Table-2:** *In vivo* evaluation of protective efficacy of anti-*Brucella* serum in mice model as homologus passive protection assay.

Group (n=6)	Transformation value of spleen count Y=log (X^#^/log X)						Mean±SD	Protective index Y_control__Y_vaccinated_

	Mice 1	Mice 2	Mice 3	Mice 4	Mice 5	Mice 6		
Control	4.253	4.265	4.125	4.325	4.282	4.313	4.260±0.071^ac^	Nil
Anti-*Brucella* serum (50 IU)	3.055	0.854	3.050	3.382	3.283	3.620	2.874±1.012^b^	1.386
Anti-*Brucella* serum (25 IU)	3.939	3.970	3.657	3.575	2.677	Died	3.564±0.524^bc^	0.696

Mean value bearing different superscripts (a, b, c,) in each column differ significantly (p<0.05).

# Total viable count of *Brucella abortus* S544 obtained from the mouse spleen. SD=Standard deviation

**Table-3:** *In vivo* evaluation of the protective efficacy of anti-*Brucella* serum in guinea pig model as heterologous passive protection assay.

Group (n=2)	Transformation value of spleen count (Y)		Mean±SD	Protective index

	Guinea pig 1	Guinea pig 2		
Control	3.540	3.952	3.746±0.291^a^	Nil
Anti-*Brucella* serum (50 IU)	3.060	2.840	2.950±0.155^a^	0.796
Anti-*Brucella* serum (25 IU)	3.366	3.304	3.335±0.043^a^	0.411

Mean value bearing similar superscripts (a) in each column differ non-significantly (p>0.05). SD=Standard deviation

**Figure-2:**
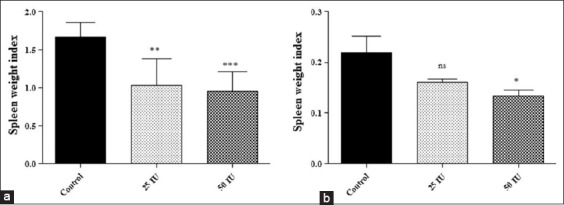
Spleen weight index of mice (a) and guinea pig (b). Data are presented as mean±standard deviation. NS=Not-significant, (*)=p<0.05, (**)=p<0.01, (***)=p<0.001.

The mean SWI in control group was significantly higher than passively immunized group either mice or guinea pig. In mice, it was 0.951±0.26, 1.03±0.34, and 1.67±0.18 for 50 IU, 25 IU passively protected, and control group, respectively, while in guinea pig, 0.133±0.011, 0.161±0.005, and 0.219±0.032 for 50 IU, 25 IU passively protected, and control group, respectively (Figure-2). Splenomegaly indicates the inability of mice/guinea pig to clear challenge bacteria and hence less protected.

## Discussion

Brucellosis in livestock is one of the serious infectious diseases that impart significant economic burden and deleterious health hazards in human. Vaccination in conjunction with strict surveillance is the only effective way to control the disease. The proper understanding of the protective immune response is the key step to develop a potent vaccine. In case of brucellosis, humoral immune response is often neglected even though it is a facultative intracellular microbe and remains extracellularly during many phases of life cycle such as just after infection, bacteremia phase, and cell-to-cell spread. Previously reported passive protection assay involving CD4+ and CD8+ or whole T-cell populations from immunized mice to native mice could not be able to conclude the exclusive role of CMI in clinical protection against virulent challenge [15]. In contrast to this, many studies involving passive transfer of specific anti-*Brucella* antibodies to naïve mice could be able to protect it against virulent challenge [22,23]. Therefore, the theme of the study was to evaluate the *in vivo* protective efficacy of *Brucella*-specific antibodies in homologous and heterologous animal model.

In our study, a dose-dependent protective response was observed in both experimental models. The PI of mice passively immunized with 50 IU or 25 IU antibodies was 1.38 and 0.69, respectively. In guinea pigs, however, animals passively immunized with 50 IU or 25 IU antibodies showed PI equivalent to 0.79 or 0.41, respectively. The higher value of PI in mice than guinea pig can be justified by the fact that mice antibodies in guinea pig perceived as foreign elicit an immune response and finally eliminated from host body. Furthermore, the significant reduction in spleen colonization was seen in passively protected mice or guinea pig that indirectly links the role of anti-*Brucella* antibodies to combat against virulent strain through opsonophagocytosis coupled with enhanced post-phagocytosis clearance [24,25]. The observations support our hypothesis that the presence of antibodies inhibits initial multiplication and eventual colonization of systemic organs by *B. abortus*. This may primarily be associated with protection against the clinical syndrome in natural hosts. The reason for a comparatively lower protective effect by the mice antibodies in guinea pig may be related to anti-murine antibody response of the animals.

Our findings are in complete concordance with previous reports that suggest the protective role of *B. abortus* polysaccharide and protein antigen-specific immune serum in murine brucellosis [16]. They speculated that immune serum can prevent dissemination of systemic infection through efficient killing of internalized *Brucella* and attributed large part of vaccinal immunity. Araya *et al*. [15] also marked the anti-lipopolysaccharides antibodies as a potent immune factor to chase the virulent *B. abortus* in mice through passive immunization. Despite the series of previous reports about the role of anti-*Brucella* antibodies to fight against field challenge, CMI is considered as a major protective mechanism against *Brucella* infection. The main lacuna is the controversial role of antibodies in disease establishment rather than considering its protective role. As reported long back, high IgG titer mostly seen in acute brucellosis prevents complement-mediated extracellular bacterial lysis and provides a bridge to establish an intracellular niche and subsequently extension of disease in cattle [26]. Although it is speculated through passive protection assay in mice that both T lymphocytes and antibodies are essential to provide complete protection, still latter one play substantially a greater role [27,28]. Recently, Adone *et al*. [29] have also concluded that specific circulating antibodies were more efficiently able to control the early dissemination and spread of *Brucella* in mice following challenge by intraperitoneal injection. In parallel line, Vitry *et al*. [30] have also speculated that antibodies alone were critical for the development of sterilizing immunity in the spleen at 50-day post-challenge. However, they considered that both CMI and humoral are equally essential for complete protection against *B. melitensis*. The other clue for a protective role of antibodies is seen in bovine brucellosis as abortion occurs mostly in the first pregnancy following infection and becomes uncommon in subsequent pregnancies because of sustained immunity [31]. Dorneles *et al*. [32] observed the predominance of IgG1 type antibodies both in S19 and RB51 vaccination regimens in cattle though it is strongly associated to a Th2 response. However, in their study, this result was in contrast to the profile observed in CMI which was predominantly Th1. Hence, this opposite finding sharply pointed out the protective enrollment of antibodies in the establishment of *Brucella* infection. Furthermore, it is the coordinated immune network that usually demands a balance between Th1 and Th2 response.

## Conclusion

Passive protection assay either in homologous or heterologous pattern anti-*Brucella* antibodies alone was able to inhibit the initial establishment of intracellular niche, which is the most valuable phase of pathogenesis and acts as deciding factor for the outcome of infection. Although the anti-*Brucella* antibodies provide a bridge for enhanced uptake by professional phagocytes, at the same time, these specific antibodies marked the virulent field strain for effective post-phagocyte clearance and significantly reduced the *B. abortus* proliferation in host body. Hence, to design any vaccine or therapeutic against *B. abortus*, humoral approach should be encouraged rather than focusing only CMI as, in usual practice, humoral arm of immunity is often neglected.

## Authors’ Contributions

MR designed the study and SV performed research experiment. SK and GM carried out the statistical analysis. SQ and AKT collaborated in writing, revising, and improvement of the article for publication. All authors read and approved the final manuscript.

## References

[1] Manish K.P, Rajesh C, Teena R, Sunil K (2013). Brucellosis-an updated review of the disease. Ind. J. Anim. Sci.

[2] World Health Organization (2014). The Control of Neglected Zoonotic Diseases.

[3] Rubach M.P, Halliday J.E, Cleaveland S, Crump J.A (2013). Brucellosis in low-income and middle-income countries. Curr. Opin. Infect. Dis.

[4] Ahmed W, Zheng K, Liu Z.F (2016). Establishment of chronic infection:Brucella's stealth strategy. Front. Cell Infect. Microbiol.

[5] Kose S, Serin S.S, Akkoclu G, Kuzucu L, Ulu Y, Ersan G, Oguz F (2014). Clinical manifestations, complications, and treatment of brucellosis:Evaluation of 72 cases. Turk. J. Med. Sci.

[6] Roushan M.R.H, Ebrahimpour S (2015). Human brucellosis:An overview. Caspian J. Intern. Med.

[7] Goodwin Z.I, Pascual D.W (2016). Brucellosis vaccines for livestock. Vet. Immunol. Immunopathol.

[8] Moreno E (2014). Retrospective and prospective perspectives on zoonotic brucellosis. Front. Microbiol.

[9] Jain L, Rawat M, Prajapati A, Tiwari A.K, Kumar B, Chaturvedi V.K, Saxena H.M, Ramakrishnan S, Kumar J, Kerketta P (2015). Protective immune-response of aluminium hydroxide gel adjuvanted phage lysate of *Brucella abortus* S19 in mice against direct virulent challenge with *B. abortus*544. Biologicals.

[10] Xavier M.N, Winter M.G, Spees A.M, Nguyen K, Atluri V.L, Silva T.M, Baumler A.J, Muller W, Santos R.L, Tsolis R.M (2013). CD4+T cell-derived IL-10 promotes *Brucella abortus* persistence via modulation of macrophage function. PLoS Pathog.

[11] Gomez G, Adams L.G, Ficht A.R, Ficht T.A (2013). Host-*Brucella* interactions and the *Brucella* genome as tools for subunit antigen discovery and immunization against brucellosis. Front. Cell Infect. Microbiol.

[12] Titball R.W (2008). Vaccines against intracellular bacterial pathogens. Drug Discov. Today.

[13] Casadevall A, Pirofski L.A (2006). A reappraisal of humoral immunity based on mechanisms of antibody-mediated protection against intracellular pathogens. Adv. Immunol.

[14] Casadevall A, Pirofski L.A (2011). A new synthesis for antibody-mediated immunity. Nat. Immunol.

[15] Araya L.N, Elzer P.H, Rowe G.E, Enright F.M, Winter A.J (1989). Temporal development of protective cell-mediated and humoral immunity in BALB/c mice infected with *Brucella abortus*. J. Immunol.

[16] Plommet M, Plommet A (1983). Immune serum-mediated effects on brucellosis evolution in mice. Infect. Immun.

[17] Indian Pharmacopoeia Commission (2014). Government of India, Ministry of Health and Family Welfare.

[18] OIE (2008). Bovine brucellosis. OIE Manual of Diagnostic Tests and Vaccines for Terrestrial Animals.

[19] Alton G.G, Jones L.M, Pietz D.E (1975). Laboratory techniques in brucellosis. Monogr. Ser. World Health Organ.

[20] Westphal O, Jann K (1965). Bacterial lipopolysaccharides extraction with phenol-water and further applications of the procedure. Meth. Carbohydr. Chem.

[21] Briggs D.J, Skeeles J.K (1984). An enzyme-linked immunosorbent assay for detecting antibodies to *Pasteurella multocida* in chickens. Avian Dis.

[22] Sulitzeanu D (1965). Mechanism of immunity against *Brucella*. Nature.

[23] Montaraz J.A, Winter A.J (1986). Comparison of living and nonliving vaccines for *Brucella abortus* in BALB/c Mice. Infect. Immun.

[24] Canning P.C, Deyoe B.L, Roth J.A (1988). Opsonin-dependent stimulation of bovine neutrophil oxidative metabolism by *Brucella abortus*. Am. J. Vet. Res.

[25] Winter A.J, Duncan J.R, Santisteban C.G, Douglas J.T, Adams L.G (1989). Capacity of passively administered antibody to prevent establishment of *Brucella abortus* infection in mice. Infect. Immun.

[26] Hoffmann E.M, Houle J.J (1995). Contradictory roles for antibody and complement in the interaction of *Brucella abortus* with its host. Crit. Rev. Microbiol.

[27] Ko J, Splitter G.A (2003). Molecular host-pathogen interaction in brucellosis:Current understanding and future approaches to vaccine development for mice and humans. Clin. Microbiol. Rev.

[28] Perkins S.D, Smither S.J, Atkins H.S (2010). Towards a *Brucella* vaccine for humans. FEMS Microbiol. Rev.

[29] Adone R, Francia M, Pistoia C, Petrucci P, Pesciaroli M, Pasquali P (2012). Protective role of antibodies induced by *Brucella melitensis* B115 against *B. melitensis* and *Brucella abortus* infections in mice. Vaccine.

[30] Vitry M.A, Mambres D.H, Trez C.D, Akira S, Ryffel B, Letesson J.J, Muraille E (2014). Humoral immunity and CD4+Th1 cells are both necessary for a fully protective immune response upon secondary infection with *Brucella melitensis*. J. Immunol.

[31] Ducrotoya M, Bertub W.J, Matopec G, Cadmusd S, Conde-Álvareze R, Gusib A.M, Welburna S, Ocholib R, Blascof J.M, Moriyóne I (2017). Brucellosis in Sub-Saharan Africa:Current challenges for management, diagnosis and control. Acta Trop.

[32] Dorneles E.M.S, Lima G.K, Teixeira-Carvalho A, Araújo M.S.S, Martins-Filho O.A, Sriranganathan N, Qublan H.A, Heinemann M.B, Lage A.P (2015). Immune response of calves vaccinated with *Brucella abortus* S19 or RB51 and revaccinated with RB51. PLoS One.

